# High-flow nasal cannula oxygen therapy versus conventional oxygen therapy in patients after planned extubation: a systematic review and meta-analysis

**DOI:** 10.1186/s13054-019-2465-y

**Published:** 2019-05-17

**Authors:** Youfeng Zhu, Haiyan Yin, Rui Zhang, Xiaoling Ye, Jianrui Wei

**Affiliations:** 1 Department of Intensive Care Unit, Guangzhou Red Cross Hospital, Medical College, Jinan University, Tongfuzhong Road No. 396, Guangzhou, 510220 Guangdong China; 2 Department of Cardiology, Guangzhou Red Cross Hospital, Medical College, Jinan University, Tongfuzhong Road No. 396, Guangzhou, 510220 Guangdong China

**Keywords:** High-flow nasal cannula, Oxygen therapy, Mechanical ventilation, Postextubation

## Abstract

**Background:**

The effect of high-flow nasal cannula (HFNC) therapy in patients after planned extubation remains inconclusive. We aimed to perform a rigorous and comprehensive systematic meta-analysis to robustly quantify the benefits of HFNC for patients after planned extubation by investigating postextubation respiratory failure and other outcomes.

**Method:**

We searched MEDLINE, EMBASE, Web of Science, and the Cochrane Library from inception to August 2018. Two researchers screened studies and collected the data independently. Randomized controlled trials (RCTs) and crossover studies were included. The main outcome was postextubation respiratory failure.

**Results:**

Ten studies (seven RCTs and three crossover studies; HFNC group: 856 patients; Conventional oxygen therapy (COT) group: 852 patients) were included. Compared with COT, HFNC may significantly reduce postextubation respiratory failure (RR, 0.61; 95% CI, 0.41, 0.92; *z* = 2.38; *P* = 0.02) and respiratory rates (standardized mean differences (SMD), − 0.70; 95% CI, − 1.16, − 0.25; *z* = 3.03; *P* = 0.002) and increase PaO_2_ (SMD, 0.30; 95% CI, 0.04, 0.56; *z* = 2.23; *P* = 0.03). There were no significant differences in reintubation rate, length of ICU and hospital stay, comfort score, PaCO_2_, mortality in ICU and hospital, and severe adverse events between HFNC and COT group.

**Conclusions:**

Our meta-analysis demonstrated that compared with COT, HFNC may significantly reduce postextubation respiratory failure and respiratory rates, increase PaO_2_, and be safely administered in patients after planned extubation. Further large-scale, multicenter studies are needed to confirm our results.

**Electronic supplementary material:**

The online version of this article (10.1186/s13054-019-2465-y) contains supplementary material, which is available to authorized users.

## Background

Mechanical ventilation is associated with significant complications that are time-dependent in nature, with a longer duration of intubation resulting in a higher incidence of complications, such as ventilator-associated pneumonia (VAP) and increased mortality [1]. Extubation is beneficial in that it decreases the risk for VAP, eliminates the work of breathing imposed by the endotracheal tube, and improves patient comfort [2].

However, after extubation, functional residual capacity which was maintained by positive end-expiratory pressure (PEEP) in invasive ventilation duration might decrease rapidly, leading to hypoxemia and extubation failure. Extubation failure, which is often defined as the need for reintubation within 24–72 h after a planned extubation, is frequent, with rates of 10–20% [3–5]. Furthermore, extubation failure is associated with an overall increase in the duration of mechanical ventilation, a greater need for tracheostomy, higher medical costs, and an increased mortality [5–7].

Conventional oxygen therapy (COT) is the main supportive treatment administered to patients after planned extubation and has conventionally been delivered using nasal prongs, cannula or masks. However, the maximal oxygen flow rates that these devices can deliver are limited. The maximal oxygen flow rate delivered by COT is only 15 L/min, which is far lower than the demands of postextubation patients with acute respiratory failure [8]. Therefore, ambient air dilutes the supplied oxygen, and finally, the fraction of inspired oxygen (FiO_2_) is significantly reduced in the alveoli. Furthermore, with oxygen delivered by COT, it is difficult to meet the requirements of heating and humidification in these patients [9]..

High-flow nasal cannula (HFNC) can supply a mixture of air and oxygen via a heated and humidified circuit at a very high flow. It can provide almost pure oxygen with a FiO_2_ of approximately 100% and a maximal flow rate up to 60 L/min [8]. The use of a HFNC may generate a positive airway pressure, ameliorate oxygenation and dyspnea, reduce the respiratory rate and work of breathing, and improve comfort [8, 10–16].

However, the effect of HFNC therapy in patients after planned extubation remains inconclusive. Some studies demonstrate that HFNC after extubation can reduce the requirement for escalation of the respiratory support, result in better oxygenation [17, 18], and be associated with better comfort and a lower reintubation rate [12]. However, in the study by Corley and colleague, HFNC therapy did not show an improvement in respiratory function in patients after planned extubation with a body mass index (BMI) ≥ 30 kg/m^2^ [19].

Therefore, we aimed to perform a rigorous and comprehensive systematic meta-analysis to robustly quantify the benefits of HFNC for patients after planned extubation by investigating postextubation respiratory failure and other outcomes.

## Methods

We performed this study in accordance with the Preferred Reporting Items for Systematic Reviews and Meta-Analyses (PRISMA) statement [20] and guidelines described in the Cochrane Handbook for Systematic Reviews of Interventions [21].

### Study selection criteria

#### Types of studies

Randomized controlled studies and crossover studies comparing HFNC and COT in the treatment of patients after planned extubation were included.

The exclusion criteria were case reports, animal studies, preclinical studies, or patients younger than 18 years.

#### Types of participants

Adult patients, who had undergone mechanical ventilation in the hospital or intensive care unit (ICU) and had planned extubation, were involved.

#### Types of interventions

Patients in the control group and intervention group received COT and HFNC therapy after extubation, respectively.

#### Types of outcome measures

Our primary outcome was postextubation respiratory failure, and the secondary outcomes included the following variables: reintubation, mortality in ICU and hospital, length of ICU and hospital stay, comfort score, respiratory rate, partial pressure of arterial oxygen (PaO_2_), partial pressure of arterial carbon dioxide (PaCO_2_), PaO_2_/FiO_2_, and severe adverse events. Postextubation respiratory failure was defined as PaO_2_/FiO_2_ < 300mmHg, hypoxemia (PaO_2_ < 60 mmHg or SpO_2_ < 90% with FiO_2_ ≥ 0.5), respiratory acidosis (pH < 7.35 and PaCO_2_ > 45 mmHg), signs of respiratory muscle fatigue and/or tachypnea > 35 breaths/min, low level of consciousness, or agitation during treatment period [22, 23]. Severe adverse events were defined as respiratory pauses with loss of consciousness, severe unstable hemodynamics, and cardiac or respiratory arrest.

### Data sources and search strategy

We searched EMBASE, MEDLINE, Web of Science, and the Cochrane Library from inception to August 2018. We also reviewed the references of relevant articles to avoid missing any studies. The details of the search strategy are shown in the Additional file 1: Appendix 1. There were no limitations on gender, patient sample size, or language.

### Data extraction

Two researchers (XLY and RZ) independently and repetitively screened titles and abstracts to evaluate the potential studies. Disagreements were resolved by consensus or by discussion with a third author (JRW). For the included studies, a full-text review was performed. Detailed study information, interventions, controls, and outcomes were extracted using a standardized data extraction form.

### Quality assessment

The qualities of the included randomized studies were assessed by modified Jadad scores, with scores of 1–3 and 4–7 judged as low and high quality, respectively. Furthermore, the included studies were evaluated for the risk of bias according to the methods described in the Cochrane Handbook [21].

### Statistical analysis

Our meta-analysis was performed on an intention-to-treat basis and involved all patients who were assigned to any study group. Data were obtained by direct extraction or by indirect calculation. For studies that reported data with cartograms, we extracted data with DigitizeIt software (Braunschweig, Germany).

For binary outcomes, we calculated the risk ratios (RRs) and 95% confidence intervals (CIs). For continuous outcomes, the standardized mean differences (SMDs) and 95% CI were calculated. We graphically displayed the outcomes by forest plots and visually inspected the potential publication bias with a funnel plot.

We used DerSimonian-Laird random effects models for pooling outcomes. The Mantel-Haenszel model was used for assessment of heterogeneity, with *P* < 0.05 and *I*^2^ > 50% indicating significant heterogeneity, and *I*^2^ > 25% indicating moderate heterogeneity.

We performed prespecified subgroup analyses for the postextubation respiratory outcomes, including study types, HFNC duration, HFNC flow, severity of patients, hypercapnic or not, and post cardiac surgery or not.

We also used trial sequential analysis to estimate the reliability of our meta-analysis by examining for sufficient data to avoid type I (false-positive) and type II (false-negative) errors. Trial sequential analysis was performed using TSA software (version 0.9.5.9 Beta; Copenhagen Trial Unit, Copenhagen, Denmark). The Lan-DeMets approach was used for construction of the O’Brien-Fleming monitoring boundaries and the optimal information size, which was set to an alpha of 0.05 with a two-sided beta of 0.80 and relative risk reduction of 20%.

### Sensitivity analysis

Sensitivity analyses were conducted on the primary outcomes to test the robustness of the results by the following methods: changing to a fixed-effect model, changing to use of the Sidik-Jonkman method for random effects, shifting to the Biggerstaff-Tweedie method, excluding any estimated values, excluding crossover studies, and excluding studies with an early termination and/or high risk of bias.

We used Review Manager Software (Version 5.3, The Cochrane Collaboration, Copenhagen, Denmark) and TSA software (version 0.9.5.9 Beta, Copenhagen Trial Unit, Copenhagen, Denmark) to conduct the statistical analysis. The Grading of Recommendations Assessment, Development, and Evaluation (GRADE) Guideline Development Tool (GRADEpro; McMaster University 2014, Hamilton, Canada) was used to evaluate the quality of evidence for each outcome [24]. The quality of evidence was stratified into four grades: high, moderate, low, or very low. *P* < 0.05 was considered statistically significant.

### Role of the funding source

This study was supported by the National Natural Science Foundation of China (NSFC81871585) and the Natural Science Foundation of Guangdong Province (2018A030313058). The study sponsors did not involve in study design, collection, data analysis and interpretation, writing of the report, or decision to submit the paper for publication.

## Results

**Fig. 1:**
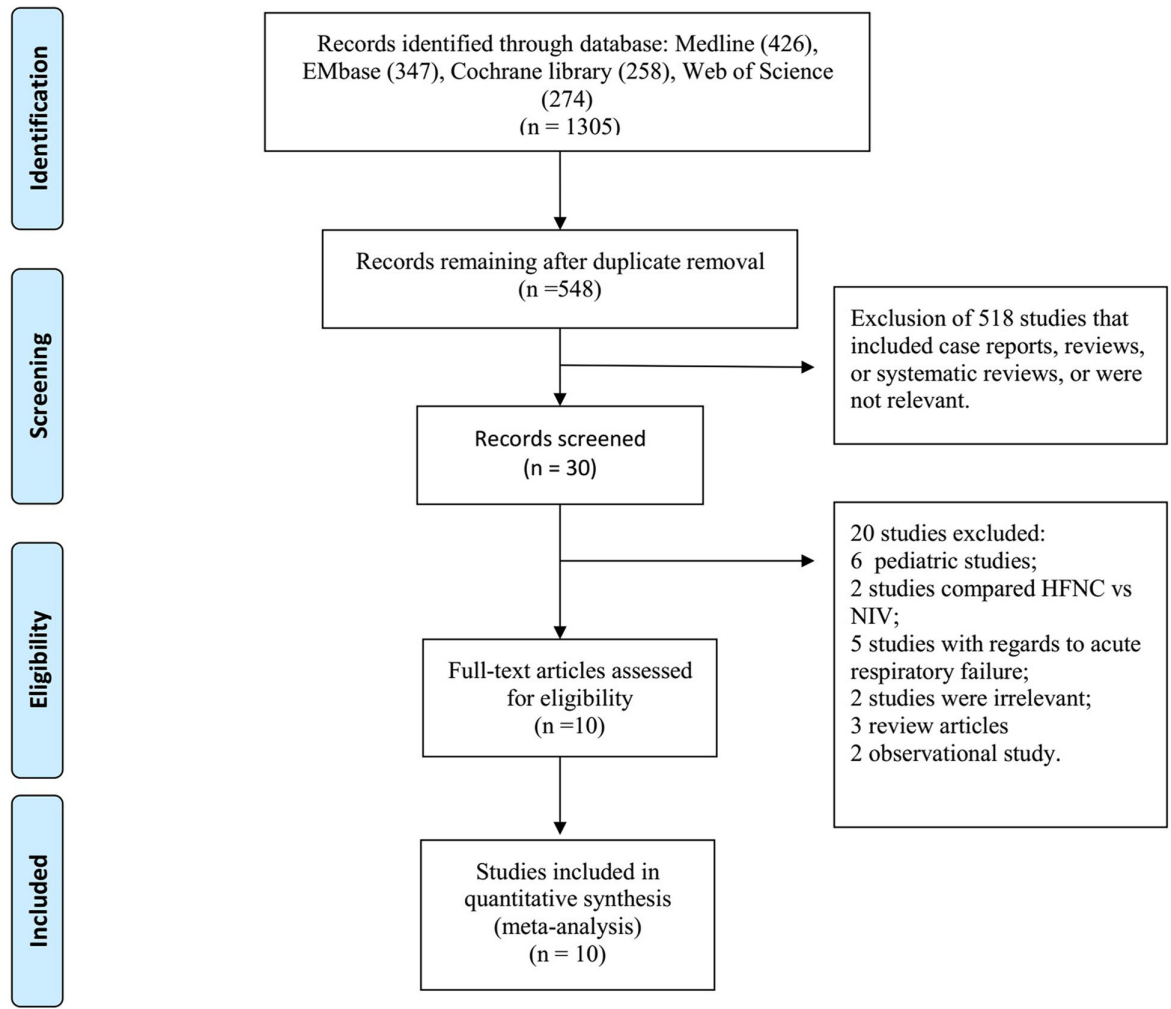
PRISMA flow diagram of the study selection process

Our study identified 1305 relevant publications. After removing duplicate results and screening the titles and abstracts, 548 publications were rescreened for titles and abstracts. Thirty studies were obtained for full-context review, and 20 studies were excluded. The details of the excluded studies and reasons for their exclusion are shown in the Additional file 1: Table S1. Finally, we included 10 studies (7 randomized controlled studies, 3 crossover studies) [12, 13, 17–19, 22, 23, 25–27] with a total of 1708 patients (median, 130 patients; range, 28–527 patients; ECMO group, 856 patients; MV group, 852 patients) in this meta-analysis. The selection process of the eligible studies is shown in Fig. 1. Of the ten included studies, 66.5% (range 47.6–85.7%; IQR 56.5%–74.1%) were men and 33.4% (range 14.3–52.4%; IQR 25.9–43.5%) were women. The durations and flow rates of HFNC in each study are shown in Table 1. The included study characteristics and baseline patient characteristics are shown in Tables 1 and 2.

**Table 1:** Basic characteristics of the included studies

Study	Study type	Country	Settings	Patients	Conventional oxygen therapy group		HFNC group					Follow-up duration
					Delivery method	Oxygen flow (L/min)	Delivery device	Humidifier	Flow rate (L/min)	FiO_2_	Duration (hours)	
Parke 2013 [17, 28]	Single-center, open-label RCT	New Zealand	ICU	Post-cardiac surgery	Face mask or nasal prongs	2–4	Optiflow™ system	AIRVO™	45	Adjusted by medical staff to maintain a SpO_2_ > 93%	48	28 days
Maggiore 2014 [12]	Two-center, open-label RCT	Italy	ICU	Medical, Surgical-trauma	Venturi mask	Adjusted to maintain an SaO_2_ 92–98%^#^	Optiflow™ system	Na	50	Adjusted to maintain an SaO_2_ 92–98%^#^	48	48 h
Corley 2015 [19]	Single-center RCT	Australia	ICU	Post-cardiac surgery with BMI ≥ 30	Face mask or nasal cannulae	2–6	Optiflow™ system	MR850 heated humidifier	35–50	Adjusted to maintain a SpO_2_ ≥ 95%	> 8	Until ICU discharge
Hernández 2016 [22]	Multicenter RCT	Spain	ICU	Medical, surgical, trauma patients with low risk of reintubation^&^	Non-rebreathing facemask or nasal cannula	Adjusted to maintain SpO2 ≥ 92%	Optiflow system	N/a	> 10	Adjusted to maintain a SpO2 ≥ 92%	24	Until hospital discharge
Futier 2016 [27]	Multicenter RCT	France	ICU	Post major abdominal surgery	Face mask or nasal prongs	Adjusted to maintain SpO2 ≥ 95%	Optiflow™ system	MR850 heated humidifier	50–60	Adjusted to maintain a SpO2 ≥ 95%	< 24	Until hospital discharge
Song 2017 [18]	Single-center RCT	China	ICU	ARF patients	Air entrainment mask	0.4	PT101AZ	N/a	< 60	0.4	24	24 h
Fernandez 2017 [23]	Multicenter RCT	Spain	ICU	High risk of extubation failure with non-hypercapnic*	Face mask or nasal prongs	Adjusted to maintain SpO2 92–95%	Optifow®	N/a	40	Adjusted to maintain a SpO2 92–95%	24	Until hospital discharge
Tiruvoipati 2010 [25]	Randomized crossover trial, single center	Australia	ICU	ICU patients	Face mask	0.3–0.4	Optiflow system	N/a	30	0.3–0.4	0.5	30 min
Rittayamai 2014 [13]	Randomized crossover trial, single center	Thailand	RCU	RCU patients	Non-rebreathing mask	Adjusted to maintain SpO2 ≥ 94%	Optiflow system	N/a	35	Adjusted to maintain a SpO2 ≥ 94%	0.5	30 min
Di mussi 2018 [26]	Self-cross control study, single center	Italy	ICU	COPD	Face mask	Adjusted to maintain an SaO2 88–92%	AIRVO™ system	N/a	20–60	Adjusted to maintain an SaO2 88–92%	1	1 h

*COT* conventional oxygen therapy, *HFNC* high-flow nasal cannula, *RCT* randomized controlled trial, *ICU* intensive care unit, *PaO*_*2*_ partial pressure of arterial oxygen, *FiO*_*2*_ fraction of inspired oxygen, *ARF* acute respiratory failure, *RCU* respiratory care unit, *COPD* chronic obstructive pulmonary disease

^#^In populations with compensated hypercapnia, SaO_2_ was 88–95%

^&^Low risk of reintubation was defined as fulfilling the following criteria: simple weaning; age < 65; heart failure was not the first reason for mechanical ventilation (MV); body mass index (BMI) < 30; Acute Physiology and Chronic Health Evaluation II score < 12; no moderate-to-severe COPD; no airway patency problems; well airway clearance ability; comorbidities< 2; and no prolonged MV

*High risk of extubation failure was defined as including at least one of the following criteria: heart failure was the first reason for MV; age > 65; non-hypercapnic moderate-to-severe COPD; Acute Physiology and Chronic Health Evaluation II score > 12; BMI > 30 kg/m^2^; duration of MV > 7 days; bad airway clearance ability; spontaneous breathing trial failure> 1

**Table 2:** Characteristics and demographic parameters of patients in the included studies

Study	Sample size (*n*)	Age (years)	Gender (M/F)	BMI (kg/m^2^)	PaO_2_/FiO_2_ at extubation	Comorbidity (*n*)^a^					Endpoints
						Respiratory disease	Hypertension	Neurologic disease	Heart disease	Others	
Parke 2013 [17, 28]											
HFNC group	169	65 (19–88)*	129/40	28.4 ± 5.3	N/a	N/a	N/a	N/a	N/a	N/a	Primary outcome: oxygenation. Secondary outcomes: Atelectasis score, length of ICU and hospital stay, 28-day mortality, oxygenation indices, escalation of respiratory support, spirometry, comfort score.
COT group	171	66 (21–87)*	129/42	29.2 ± 5.5	N/a	N/a	N/a	N/a	N/a	N/a	
Maggiore 2014 [12]											
HFNC group	53	65 ± 18	33/20	N/a	239.4 ± 42.4	24	N/a	N/a	6	23	Primary outcome: oxygenation. Secondary outcomes: oxygen desaturation, device displacement, requiring ventilator support^#^, reintubation, discomfort score.
COT group	52	64 ± 17	35/17	N/a	241.7 ± 51.1	24	N/a	N/a	5	23	
Corley 2015 [19]											
HFNC group	81	63 ± 11.4	58/23	36 ± 5.2	N/a	26	N/a	N/a	N/a	5	Primary outcome: Atelectasis score. Secondary outcomes: RR, oxygenation, subjective dyspnea, reintubation, failure of allocated treatment.
COT group	74	65 ± 11.1	56/18	35 ± 4.3	N/a	20	N/a	N/a	N/a	6	
Hernández 2016 [22]											
HFNC group	264	51 ± 13.1	164/100	< 30	227 ± 25	32	43	20	20	89	Primary outcome: reintubation within 3 days. Secondary outcomes: mortality, multiorgan failure, postextubation respiratory failure, sepsis, respiratory infection, length of ICU and hospital stay, adverse events, time to reintubation.
COT group	263	51.8 ± 12.2	153/110	< 30	237 ± 34	30	37	34	23	79	
Futier 2016 [27]											
HFNC group	108	62 ± 12	61/47	25 ± 4	N/a	10	34	N/a	N/a	76	Primary outcome: rate of hypoxemia 1 h after extubation. Secondary outcomes: pulmonary complications, length of ICU and hospital stay, in-hospital mortality.
COT group	112	61 ± 13	64/48	25 ± 4	N/a	8	35	N/a	N/a	67	
Song 2017 [18]											
HFNC group	30	66 ± 14	16/14	N/a	207 ± 27.5	19	N/a	N/a	7	4	Primary outcome: therapy success rate. Secondary outcomes: RR, HR, oxygenation indices, MAP.
COT group	30	71 ± 13	18/12	N/a	204 ± 29	19	N/a	N/a	7	4	
Fernandez 2017 [23]											
HFNC group	78	67.3 ± 12	46/32	N/a	N/a	22	N/a	N/a	9	N/a	Primary outcome: respiratory failure within 3 days. Secondary outcomes: length of ICU and hospital stay, reintubation, mortality.
COT group	77	69.7 ± 13	55/22	N/a	N/a	24	N/a	N/a	9	N/a	
Tiruvoipati 2010 [25]											
HFNC group	42	65.22 ± 17.6	20/22	N/a	> 175	N/a	N/a	N/a	N/a	N/a	Primary outcome: efficacy of oxygenation. Secondary outcomes: HR, RR, blood pressure, comfort score, tolerance score.
COT group	42	65.22 ± 17.6	20/22	N/a	> 175	N/a	N/a	N/a	N/a	N/a	
Rittayamai 2014 [13]											
HFNC group	17	66.8 ± 13.8	10/7	N/a	≥ 150	9	8	N/a	8	N/a	Primary outcome: dyspnea score. Secondary outcomes: HR, RR, MAP, comfort score.
COT group	17	66.8 ± 13.8	10/7	N/a	≥ 150	9	8	N/a	8	N/a	
Di mussi 2018 [26]											
HFNC group	14	71.5 ± 9	12/2	N/a	> 150	N/a	N/a	N/a	N/a	N/a	Primary outcome: neuroventilatory drive and work of breathing. Secondary outcomes: RR, oxygenation indices.
COT group	14	71.5 ± 9	12/2	N/a	> 150	N/a	N/a	N/a	N/a	N/a	

Respiratory disease including pneumonia, chronic obstructive pulmonary disease, asthma, and other respiratory disease; heart disease including cardiogenic pulmonary edema, congestive heart failure, and cardiac arrest

*M* male, *F* female, *BMI* body mass index, *PaO*_*2*_ arterial partial pressure of oxygen, *FiO*_*2*_ fraction of the inspired oxygen, *HFNC* high-flow nasal cannula, *COT* conventional oxygen therapy, *BMI* body-mass index, *SpO*_*2*_ pulse oxygen saturation, *RR* respiratory rate, *ARR* absolute risk reduction, *HR* heart rate, *MAP* mean arterial pressure

Plus-minus values are the means ± SDs

^a^Patients can have more than 1 comorbidity

*Values are median and interquartile range

^#^Requiring ventilator support including any form of ventilator support, e.g., noninvasive ventilation or mechanical ventilation

Eight of the ten included studies were considered to be at low risk of bias as evaluated by the Cochrane risk of bias tool and modified Jadad score (Additional file 1: Figure S1 and S2, Table S2).

### Postextubation respiratory failure

**Fig. 2:**
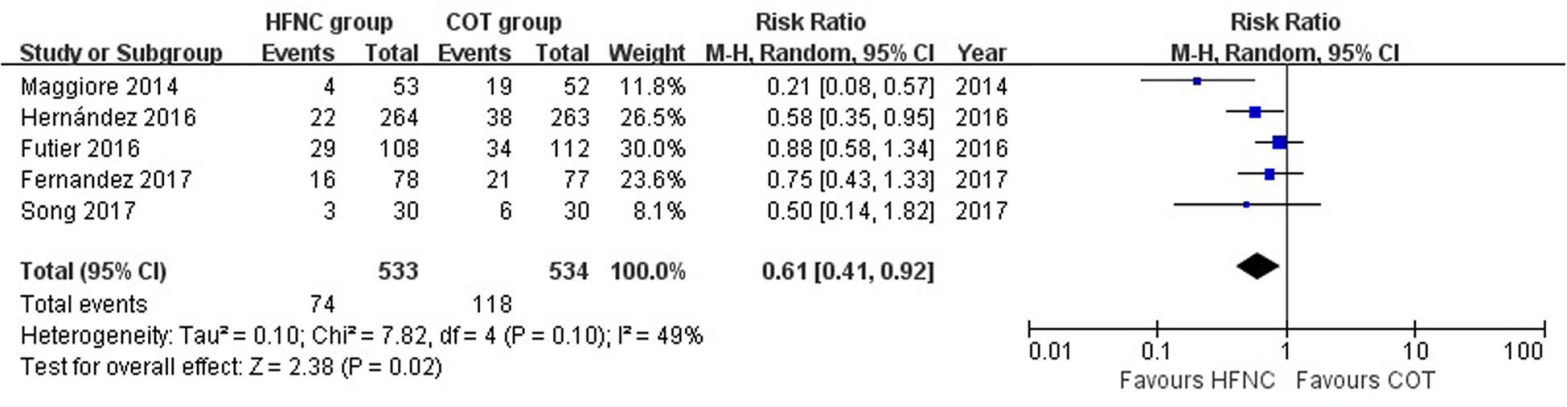
Comparison of postextubation respiratory failure between the HFNC group and COT group

The data on postextubation respiratory failure were available from five studies. When these data were pooled together, the HFNC group showed a significant reduction of postextubation respiratory failure compared with that of the COT group (RR, 0.61; 95% CI, 0.41, 0.92; *z* = 2.38; *P* = 0.02; Fig. 2). There was moderate heterogeneity among the studies (chi^2^ = 7.82, df = 4, *P* = 0.10, *I*^2^ = 49%) which might be due to a heterogeneous population of patients among the included studies (Table 1) and various treatment measures after extubation. Subgroup analyses demonstrated no significant interactions with HFNC duration (HFNC ≥ 24 h RR, 0.52 [95% CI, 0.33–0.84] vs. HFNC < 24 h RR, 0.88 [95% CI, 0.58–1.34], *P*_interaction_ = 0.10), HFNC flow (HFNC ≥ 40 L/min RR, 0.59 [95% CI, 0.34–1.05] vs. HFNC< 40 L/min RR, 0.58 [95% CI, 0.35–0.95], *P*_interaction_ = 0.94), severity of patients (severe subgroup RR, 0.42 [95% CI, 0.12–1.52] vs. non severe subgroup RR, 0.72 [95% CI, 0.53–0.99], *P*_interaction_ = 0.42), and hypercapnic or not (non-hypercapnic RR, 0.65 [95% CI, 0.44–0.94] vs. mixed subgroup RR, 0.48 [95% CI, 0.18–1.29], *P*_interaction_ = 0.59) (Table 3, Additional file 1: Figure S3-S6).

**Table 3:** Subgroup analysis for outcomes (displayed with RR or SMD)

Outcomes	Postextubation respiratory failure (RR)	Reintubation (RR)	Respiratory rate (SMD)	PaO_2_ (SMD)
Subgroup analyses				
Study type				
RCT study	0.61 (0.41, 0.92)	0.58 (0.30, 1.11)	− 1.12 (− 1.45, − 0.79)	0.39 (− 0.02,0.79)
Crossover study	Na	Na	− 0.35 (− 0.75, 0.04)	0.14 (− 0.23, 0.51)
Interaction	Na	Na	*P* = 0.004	*P* = 0.38
Severity of patients				
Severe populations	0.42 (0.12, 1.52)	0.39 (0.13, 1.19)	− 0.56 (− 1.29, 0.18)	0.31 (0.05, 0.58)
Non severe population	0.72 (0.53, 0.99)	0.81 (0.27, 2.45)	− 0.93 (− 1.36, − 0.50)	0.35 (− 0.29, 1.00)
Interaction	0.42	0.36	0.39	0.92
HFNC flow				
≥ 40 L/min	0.59 (0.34, 1.05)	0.72 (0.29, 1.83)	− 1.14 (− 1.47, − 0.81)	0.39 (− 0.02, 0.79)
< 40 L/min	0.58 (0.35, 0.95)	0.39 (0.21, 0.72)	− 0.44 (− 1.09, 0.21)	0.10 (− 0.33, 0.52)
Interaction	0.94	0.28	0.06	0.33
Non-hypercapnic or not				
Non-hypercapnic	0.65 (0.44, 0.94)	0.52 (0.29, 0.93)	− 0.16 (− 0.59, 0.26)	0.10 (− 0.33, 0.52)
Mixed^a^	0.48 (0.18, 1.29)	0.45 (0.12, 1.77)	− 1.07 (− 1.37, − 0.77)	0.39 (− 0.02, 0.79)
Interaction	0.59	0.86	0.0007	0.33
HFNC duration				
≥ 24 h	0.52 (0.33, 0.84)	0.48 (0.26, 0.89)	− 1.12 (− 1.45, − 0.79)	0.58 (0.27, 0.90)
< 24 h	0.88 (0.58, 1.34)	0.88 (0.11, 7.33)	− 0.35 (− 0.75, 0.04)	0.09 (− 0.13, 0.30)
Interaction	*P* = 0.10	*P* = 0.59	*P* = 0.004	*P* = 0.01
Post cardiac surgery or not				
Post cardiac surgery	Na	0.96 (0.04, 24.84)	Na	Na
Other patients	0.62 (0.42, 0.92)	0.55 (0.28, 1.08)	− 0.70 (− 1.16, − 0.25)	0.30 (0.04, 0.56)
Interaction	Na	*P* = 0.74	Na	Na

RRs and 95% confidence intervals (CIs) were calculated for the binary data, and the standardized mean differences (SMDs) and 95% CIs were calculated for the continuous data variables

*RR* risk ratio, *SMD* standardized mean difference, *RCT* randomized controlled trial, *HFNC* high-flow nasal cannula, *PaO*_*2*_ partial pressure of arterial oxygen

^a^Means studies included patients with hypoxemic or/and hypercapnic respiratory failure

The result was robust to multiple sensitivity analyses, including changing to a fixed-effect model, SJ effect model, or BT effect model, excluding any estimated values, excluding crossover studies, or excluding the high-risk bias study and/or early termination study (Additional file 1: Table S3). For primary outcome, trial sequential analysis confirmed that the required information size was not reached; however, the *Z*-curve had crossed O’Brian-Fleming monitoring boundaries, indicating that HFNC was beneficial than COT in postextubation respiratory failure (Additional file 1: Figure S7).

**Fig. 3:**
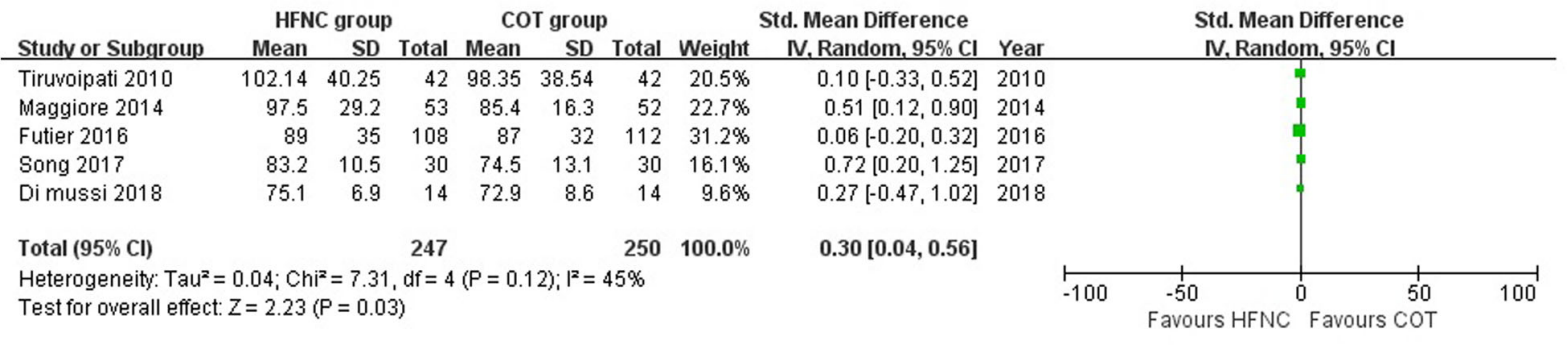
Comparison of PaO_2_ between the HFNC group and COT group

**Fig. 4:**
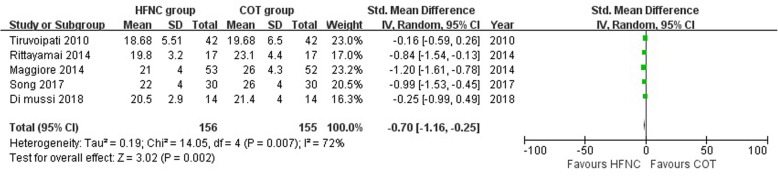
Comparison of respiratory rates between the HFNC group and COT group=

PaO_2_ was significantly higher with HFNC compared with COT (SMD, 0.30; 95% CI, 0.04, 0.56; *z* = 2.23; *P* = 0.03; Fig. 3), and respiratory rate was significantly lower in HFNC group compared with COT group (SMD, − 0.70; 95% CI, − 1.16, − 0.25; *z* = 3.03; *P* = 0.002; Fig. 4). There were moderate to high heterogeneity which might be due to a heterogeneous population of patients among the included studies (Table 1). Sensitivity analyses did not change the overall findings (Additional file 1: Table S3). Subgroup analyses demonstrated there were significant interactions with regard to study type (RCT study SMD, − 1.12 [95% CI, − 1.45, − 0.79] vs. Crossover study, − 0.35 [95% CI, − 0.75, 0.04], *P*_interaction_ = 0.004), HFNC duration (HFNC ≥ 24 h SMD, − 1.12 [95% CI, − 1.45, − 0.79] vs. HFNC < 24 h SMD, − 0.35 [95% CI, − 0.75, 0.04], *P*_interaction_ = 0.004), hypercapnic or not (non-hypercapnic SMD, − 0.16 [95% CI, − 0.59,0.26] vs. mixed subgroup SMD, − 1.07 [95% CI, − 1.37, − 0.77], *P*_interaction_ = 0.007) in respiratory rate (Table 3).

There were no significant differences in reintubation rate, length of ICU and hospital stay, comfort score, PaCO_2_, and mortality in ICU and hospital between HFNC and COT group (Additional file 1: Figure S8-S14) .

### Severe adverse events

Among the included studies, no severe adverse effects were reported in both groups.

Visual inspection of the funnel plot did not show any publication bias (Additional file 1: Figure S15–S24). Summary of findings with GRADE system are shown in Table 4.

**Table 4:** Summary of findings

Outcomes	Anticipated absolute effects^*^ (95% CI)		Relative effect (95% CI)	No. of participants (studies)	Certainty of the evidence (GRADE)	Comments
	Risk with COT	Risk with HFNC				
Postextubation respiratory failure	219 per 1000	136 per 1000 (92 to 202)	RR 0.62 (0.42 to 0.92)	1067 (5 RCTs)	⨁⨁⨁⨁ High	
PaO_2_ (mmHg)	The mean paO_2_ was 83.63 mmHg	The mean paO2 in the intervention group was 89.39 mmHg (75.91 to 102.86 mmHg)	–	497 (5 RCTs)	⨁⨁⨁⨁ High	
Respiratory rates (breaths per minute)	The mean respiratory rates was 23.24 breaths per minute	The mean respiratory rates in the intervention group was 20.4 breaths per minute (18.84 to 21.95 breaths per minute)	–	311 (5 RCTs)	⨁⨁⨁⨁ High	Respiratory rates obtained from the study by Maggiore and colleague was reported with cartograms, and we extracted data with DigitizeIt software (Braunschweig, Germany).
Reintubation	82 per 1000	48 per 1000 (25 to 91)	RR 0.58 (0.30 to 1.11)	1562 (7 RCTs)	⨁⨁⨁⨁ High	

Patient or population: patients after planned extubation

Setting:

Intervention: HFNC

Comparison: COT

GRADE Working Group grades of evidence:

High certainty: We are very confident that the true effect lies close to that of the estimate of the effect

Moderate certainty: We are moderately confident in the effect estimate—the true effect is likely to be close to the estimate of the effect, but there is a possibility that it is substantially different

Low certainty: Our confidence in the effect estimate is limited—the true effect may be substantially different from the estimate of the effect

Very low certainty: We have very little confidence in the effect estimate—the true effect is likely to be substantially different from the estimate of effect

*The risk in the intervention group (and its 95% confidence interval) is based on the assumed risk in the comparison group and the relative effect of the intervention (and its 95% CI)

*CI* confidence interval, *RR* risk ratio

## Discussion

This systematic review and meta-analysis, including 1708 planned patients (HFNC group: 856 patients; COT group: 852 patients), demonstrated that compared with COT, HFNC might significantly reduce postextubation respiratory failure (RR, 0.61; 95% CI, 0.41, 0.92; *z* = 2.38; *P* = 0.02) and respiratory rates (SMD, − 0.70; 95% CI, − 1.16, − 0.25; *z* = 3.02; *P* = 0.002) and increase PaO_2_ (SMD, 0.30; 95% CI, 0.04, 0.56; *z* = 2.23; *P* = 0.03). There were no significant differences in reintubation rate, length of ICU and hospital stay, comfort score, PaCO_2_, mortality in ICU and hospital, and severe adverse events between the HFNC and COT group.

The present systematic review and meta-analysis demonstrated a biologically plausible association between HFNC therapy and decreased postextubation respiratory failure in planned extubation patients. Previous animal and human mechanistic studies have demonstrated that HFNC enables to deliver more adequate inspiratory flow, flush the nasopharyngeal dead space, and deliver warm and humidified gas, thereby generating a positive airway pressure, ameliorating oxygenation and dyspnea, reducing the respiratory rate and work of breathing, and improving comfort [29–32].

Our study showed that HFNC might significantly reduce postextubation respiratory failure in patients after planned extubation. The result was consistent with multiple subgroup analyses, sensitivity analyses, and trial sequential analysis. However, as there was moderate heterogeneity (chi^2^ = 7.82, df = 4, *P* = 0.10, *I*^2^ = 49%) among the included studies, which might have been due to the heterogeneous population of patients and various treatment measures after extubation, a decisive conclusion should be made cautiously. Further large-scale, multicenter studies are needed to confirm our results.

A previous study showed that in patients with acute hypoxemic respiratory failure, an increasing HFNC flow rate (30, 45, and 60 L/min) progressively decreased inspiratory effort and improved lung aeration, dynamic compliance, and oxygenation [33]. In a study by Parke and coworkers, patients’ nasopharyngeal pressures were measured when HFNC was used with gas flows of 30, 40, and 50 L/min [28]. Researchers demonstrated that the mean nasopharyngeal pressures were 1.5 ± 0.6, 2.2 ± 0.8, and 3.1 ± 1.2 mmHg at 30, 40, and 50 L/min during HFNC therapy, respectively. They showed that the level of PEEP as a benefit of HFNC therapy was flow-dependent. The various starting flows may have led to different levels of PEEP and could have influenced the results. Thus, we performed a subgroup analysis according to the flow rate of HFNC (≥ 40 L/min, < 40 L/min), and we did not find significant interactions between subgroups with regard to postextubation respiratory failure (*P*_interaction_ = 0.94), reintubation (*P*_interaction_ = 0.28), respiratory rate (*P*_interaction_ = 0.06), and PaO_2_ (*P*_interaction_ = 0.33). These results may be due to the benefits of HFNC being produced not only by a high-flow rate but also through the effect of heating and humidification, reducing the work of breathing and being more comfortable for patients [34, 35].

Subgroup analysis with regard to HFNC duration showed a reduction of postextubation respiratory failure in studies that used this therapy for ≥ 24 h (RR, 0.53; 95% CI, 0.34, 0.84; *z* = 2.73; *P* = 0.006) and found no efficacy in those that used HFNC < 24 h (RR, 0.88; 95% CI, 0.58, 1.34; *z* = 0.57; *P* = 0.57). Our previous study also showed that HFNC therapy might decrease the rate of escalation of respiratory support and the intubation rate when ARF patients were treated with HFNC for ≥ 24 h [36]. However, no significant interaction (*P*_interaction_ = 0.11) was found between subgroups in the present meta-analysis. This may be due to only one study involved in the subgroup that used HFNC < 24 h [27]. Further studies comparing the effect of duration for HFNC treatment in patients after planned extubation are needed.

Patients who presented with hypoxemic or hypercapnic respiratory failure after planed extubation might lead to different results. Therefore, we performed a subgroup analysis by stratified studies into a nonhypercapnic subgroup and mixed subgroup (hypoxemic or/and hypercapnic). In the study by Parke and colleagues, the baseline PaCO_2_ levels were not reported; thus, it was difficult to know whether patients with hypercapnic respiratory failure were studied [17]. In the studies by Hernández and colleagues and by Fernandez and coworkers, nonhypercapnic patients were studied [22, 23]. In the 7 other studies, mixed patients were included [12, 13, 18, 19, 25–27]. Subgroup analysis found that there were no differences in postextubation respiratory failure (*P*_interaction_ = 0.59), reintubation (*P*_interaction_ = 0.86), and PaO_2_ (*P*_interaction_ = 0.33) between subgroups. And there was a significant difference in respiratory rate (*P*_interaction_ = 0.0007); however, this result needs to be interpreted with great caution because only one study was included in the nonhypercapnic subgroup [25].

Patients’ severity might influence the effect of HFNC. Therefore, we performed subgroup analyses according to the severity of patients among included studies. All of the included studies reported severity scores using different severity evaluation methods. Four of the included studies reported Acute Physiology And Chronic Health Evaluation (APACHE) II scores. In the study by Song and coworkers, the APACHE II scores in the COT group and HFNC group were 12.36 ± 3.29 and 12.87 ± 3.0, respectively [18]. In the study by Corley and colleagues, the APACHE II scores in the COT group and HFNC group were 15 ± 3.9 and 15 ± 3.6, respectively [19]. In the study by Hernández and colleagues, the APACHE II scores in the COT group and HFNC group were 13 (7–17) and 14 (9–16), respectively [22]. In the study by Fernandez and coworkers, the APACHE II scores in the COT group and HFNC group were 21 ± 8.2 and 21 ± 8.8, respectively [23]. In the study by Tiruvoipati and coworkers, the APACHE III scores were reported, and the scores in the protocol A group and protocol B group were 70.55 ± 27.39 and 72.95 ± 23.22, respectively [25]. Three of the included studies reported the Simplified Acute Physiology Score (SAPS) II. In the study by Maggiore and colleagues, the SAPS II scores in the COT group and HFNC group were 44 ± 16 and 43 ± 14, respectively [12]. In the crossover study by Rittayamai and coworkers, the SAPS II score was 30.9 ± 4.4 [13]. In the crossover study by Di mussi and colleagues, the SAPS II score was 39.6 ± 13.2, and the Sequential Organ Failure Assessment (SOFA) score was 5.6 ± 2.5 [26]. In the study by Parke and coworkers, the EuroSCORE was reported, and the scores in the COT group and HFNC group were 5.3 ± 2.8 and 5.1 ± 2.8, respectively [17]. In the study by Futier and colleagues, the preoperative risk score was reported; few patients in both groups (15% patients in the COT group and 17% patients in the HFNC group) were at high-risk levels, and the main patients in both groups were at moderate levels [27]. According to the severity scores of populations, we stratified the included studies into a severe subgroup (APACHE II ≥ 15, SAPS II ≥ 38, SOFA ≥ 2) and non-severe subgroup (APACHE II < 15, SAPS II < 38, SOFA < 2) [37, 38]. However, we found no interactions between subgroups with regard to postextubation respiratory failure (*P*_interaction_ = 0.42), reintubation (*P*_interaction_ = 0.36), respiratory rate (*P*_interaction_ = 0.39), and PaO_2_ (*P*_interaction_ = 0.92), which meant that the severity of patients would not influence the effect of HFNC with regard to postextubation respiratory failure, reintubation, respiratory rate, and PaO_2_.

Although a lower postextubation respiratory failure would be expected to decrease reintubation rate and shorten the length of ICU and hospital stays, no differences were found in this aspect in the present meta-analysis. This may be due to a heterogeneous population of patients included in our study and various clinical treatment measures applied when patients suffered postextubation failure. In three of the included studies, when patients in COT group need an escalation of respiratory support, HFNC therapy was applied which might make it difficult to interpret the results [17–19]. Hernández et al. speculated that this may be because the percentage of reintubated patients was too low to affect outcome variables in the entire group [22].

There are several limitations to our meta-analysis. First, this study involved a heterogeneous population of patients among the included studies (Table 1), which could affect our results. To address this problem, subgroup analyses and multiple sensitivity analysis were performed. And the subgroup results remained consistent with the overall findings. Multiple sensitivity analysis including changing effect models, excluding the high-risk bias study and/or early termination studies, did not change the overall results (Table 4). So we believed the results of our study were credible. Furthermore, the heterogeneous population of patients in our study enabled our results to have a general external validity in mixed populations of critically ill patients. Second, the duration of HFNC varied among the included studies (Table 1). Our previous study showed that HFNC therapy might decrease the rate of escalation of respiratory support and the intubation rate when ARF patients were treated with HFNC for ≥ 24 h [36]. However, a subgroup analysis of the present study did not find any interactions with regard to the duration of HFNC (Table 3). Further studies comparing the effect of duration in HFNC treatment in patients after planned extubation are needed. Third, among the included studies, FiO_2_ was titrated according to SpO_2_ or SaO_2_ (Table 1). We have reviewed all the studies included in this meta-analysis. Unexpectedly, except for the studies by Song and Tiruvoipati, the clear FiO_2_ values in these studies were not well reported. Subgroup analysis with regard to FiO_2_ was not performed. Finally, we include three crossover studies in the present study and crossover studies are limited by nature. Hence, we used the GRADE Guideline Development Tool to evaluate the quality of evidence which showed equal quality levels between crossover studies and randomized studies, showing that the results from the crossover studies should also be seriously considered.

## Conclusions

Our meta-analysis demonstrated that compared with COT therapy, HFNC therapy may significantly reduce postextubation respiratory failure and respiratory rates, may increase PaO_2_, and may be safely administered in patients after planned extubation. Further large-scale, multicenter studies are needed to confirm our results.

## Additional file

Additional file 1: **Table S1.** Studies Excluded after Full-text Review. **Table S2.** Quality of the included RCT studies. **Table S3.** Sensitivity analysis of the outcomes. **Figure S1.** Risk of bias graph. **Figure S2.** Risk of bias summary. **Figure S3.** Subgroup analysis of postextubation respiratory failure between the HFNC group and COT group according to HFNC duration. **Figure S4.** Subgroup analysis of postextubation respiratory failure between the HFNC group and COT group according to HFNC flow. **Figure S5.** Subgroup analysis of postextubation respiratory failure between the HFNC group and COT group according to severity of patients. **Figure S6.** Subgroup analysis of postextubation respiratory failure between the HFNC group and COT group according to hypercapnic or not. **Figure S7.** Trial sequential analysis. **Figure S8.** Comparison of reintubation between the two groups. **Figure S9.** Comparison of length of ICU stay between the two groups. **Figure S10.** Comparison of length of hospital stay between the two groups. **Figure S11.** Comparison of comfort score between the two groups. **Figure S12.** Comparison of PaCO_2_ between the two groups. **Figure S13.** Comparison of ICU mortality between the two groups. **Figure S14.** Comparison of hospital mortality between the two groups. **Figure S15.** Funnel plot of comparison for postextubation respiratory failure between the two group. **Figure S16.** Funnel plot of comparison for PaO_2_. **Figure S17.** Funnel plot of comparison for respiratory rates. **Figure S18.** Funnel plot of comparison for reintubation.** Figure S19.** Funnel plot of comparison for length of ICU stay. **Figure S20.** Funnel plot of comparison for length of hospital stay. **Figure S21.** Funnel plot of comparison for comfort score. **Figure S22.** Funnel plot of comparison for PaCO_2_. **Figure S23.** Funnel plot of comparison for ICU mortality. **Figure S24.** Funnel plot of comparison for hospital mortality. (DOC 613 kb)

## Abbreviations

BMI: Body mass index
COT: Conventional oxygen therapy
FiO_2_: Fraction of inspired oxygen
HFNC: High-flow nasal cannula
ICU: Intensive care unit
NIV: Noninvasive ventilation
PaO_2_: Partial pressure of oxygen in arterial blood
WMD: Weighted mean differences
