# Supplementary material for: High-flow nasal cannula oxygen therapy versus conventional oxygen therapy in patients after planned extubation: a systematic review and meta-analysis
Source: Crit Care. 2019 May 17;23:180. doi: 10.1186/s13054-019-2465-y (PMC6525416; doi:10.1186/s13054-019-2465-y)

**Supplemental files**

**Appendix 1. Details of Search Strategy**

1. **Medline**
   1. oxygen therapy. ti,ab,kw.
   2. oxygen inhalation therapy. ti,ab,kw.
   3. oxygen delivery devices. ti,ab,kw.
   4. standard oxygen. ti,ab,kw.
   5. high flow nasal cannula. ti,ab,kw.
   6. high flow oxygen therapy. ti,ab,kw.
   7. nasal high flow oxygen therapy. ti,ab,kw.
   8. nasal cannula. ti,ab,kw.
   9. 1 or 2 or 3 or 4 or 5 or 6 or 7 or 8
   10. postextubation.ti,ab,kw.
   11. extubation.ti,ab,kw, MeSH Terms.
   12. 10 or 11
   13. 9 and 12
   14. random.ti,ab,kw.
   15. randomly.ti,ab,kw.
   16. randomized.ti,ab,kw.
   17. 16 or 17 or 18
   18. 13 and 17
2. **Embase**
   1. oxygen therapy. ti,ab,kw.
   2. Oxygen inhalation therapy. ti,ab,kw.
   3. Oxygen delivery devices. ti,ab,kw.
   4. standard oxygen. ti,ab,kw.
   5. high flow nasal cannula. ti,ab,kw.
   6. high flow oxygen therapy. ti,ab,kw.
   7. nasal high flow oxygen therapy. ti,ab,kw.
   8. Nasal Cannula. ti,ab,kw.
   9. 1 or 2 or 3 or 4 or 5 or 6 or 7 or 8
   10. Postextubation respiratory failure.ti,ab,kw.
   11. extubation.ti,ab,kw.
   12. 10 or 11
   13. 9 and 12
   14. 'adult'/exp
   15. random.ti,ab,kw.
   16. randomized.ti,ab,kw.
   17. 15 or 16
   18. 14 and 17
   19. 13 and 18
3. **Cochrane Library**
   1. oxygen therapy. ti,ab,kw.
   2. Oxygen inhalation therapy. ti,ab,kw.
   3. Oxygen delivery. ti,ab,kw.
   4. standard oxygen therapy. ti,ab,kw.
   5. high flow nasal cannula. ti,ab,kw.
   6. high flow oxygen therapy. ti,ab,kw.
   7. nasal high flow oxygen therapy. ti,ab,kw.
   8. Nasal Cannula. ti,ab,kw.
   9. 1 or 2 or 3 or 4 or 5 or 6 or 7 or 8
   10. Postextubation respiratory failure.ti,ab,kw.
   11. extubation
   12. 9 and 10
   13. random.ti,ab,kw.
   14. randomly.ti,ab,kw.
   15. randomized.ti,ab,kw.
   16. 12 or 13 or 14
   17. 11 and 15
4. **Web of Science**
   1. Topic: oxygen therapy
   2. Topic: Oxygen inhalation therapy
   3. Topic: Oxygen delivery
   4. Title: high flow nasal cannula
   5. Title/Topic: high flow oxygen therapy
   6. Title/Topic: postextubation
   7. Title/Topic: extubation
   8. #1 or #2 or #3 or #4 or 5
   9. #6 or #7
   10. #8 and #9

**TableS1.Studies Excluded after Full-text Review**

| **Author, year** | **Reasons for exclusion** |
| --- | --- |
| Kamerkar,2017[1] | Paediatrics study |
| Noguchi,2017[2] | Not randomized controlled study |
| Ferguson,2017[3] | Systematic review and meta-analysis |
| Zhang,2018[4] | Observational study |
| Kamit,2018[5] | Paediatrics study |
| Shioji,2017[6] | Paediatrics study |
| Schreiber,2016[7] | Review |
| Hernández,2016[8] | HFNC vs Non-invasive ventilation |
| Arora,2012[9] | Paediatrics study |
| Nedel,2017[10] | Review |
| Lemiale,2017[11] | About respiratory failure patients |
| Frat,2014[12] | Post-hoc analysis |
| Bell,2015[13] | About respiratory failure patients |
| Frat,2015[14] | About respiratory failure patients |
| Lemiale,2015[15] | About respiratory failure patients |
| Jones,2016[16] | About respiratory failure patients |
| Coudroy,2016[17] | Observational study |
| Milani,2016[18] | Paediatrics study |
| Schmid,2017[19] | Paediatrics study |
| Stephan,2017[20] | HFNC vs Non-invasive ventilation |

**Reference:**

1. Kamerkar A, Hotz J, Morzov R, Newth CJL, Ross PA, Khemani RG. Comparison of Effort of Breathing for Infants on Nasal Modes of Respiratory Support. J Pediatr. 2017;185:26-32.e3.
2. Noguchi S, Saito J, Akaishi M, Ohta D, Hirota K. Successful High Flow Nasal Cannula Therapy in a Patient with Myotonic Dystrophy during Perioperative Period. Masui. 2017;66:303-305.
3. Ferguson KN, Roberts CT, Manley BJ, Davis PG. Interventions to Improve Rates of Successful Extubation in Preterm Infants: A Systematic Review and Meta-analysis. JAMA Pediatr. 2017;171(2):165-174.

4. Zhang JC, Wu FX, Meng LL, Zeng CY, Lu YQ. A study on the effects and safety of sequential humidified high flow nasal cannula oxygenation therapy on the COPD patients after extubation. Zhonghua Yi Xue Za Zhi. 2018;98(2):109-112.

5. Kamit Can F, Anil AB, Anil M, Zengin N, Durak F, Alparslan C, Goc Z. Predictive factors for the outcome of high flow nasal cannula therapy in a pediatric intensive care unit: Is the SpO2/FiO2 ratio useful? J Crit Care. 2018;44:436-444.

6.Shioji N, Iwasaki T, Kanazawa T, Shimizu K, Suemori T, Sugimoto K, Kuroe Y, Morimatsu H. Physiological impact of high-flow nasal cannula therapy on postextubation acute respiratory failure after pediatric cardiac surgery: a prospective observational study. J Intensive Care. 2017;5:35.

7.Schreiber A, DI Marco F, Braido F, Solidoro P. High flow nasal cannula oxygen therapy, work in progress in respiratory critical care. Minerva Med. 2016;107(6 Suppl 1):14-20.

8.Hernández G, Vaquero C, Colinas L, Cuena R, González P, Canabal A, Sanchez S, Rodriguez ML, Villasclaras A, Fernández R. Effect of Postextubation High-Flow Nasal Cannula vs Noninvasive Ventilation on Reintubation and Postextubation Respiratory Failure in High-Risk Patients: A Randomized Clinical Trial. JAMA. 2016 Oct 18;316(15):1565-1574.

9. B Arora, P Mahajan, MA Zidan, U Sethuraman. Nasopharyngeal airway pressures in bronchiolitis patients treated with high-flow nasal cannula oxygen therapy. Pediatr Emerg Care. 2012;28: 1179-1184.

10. Nedel WL, Deutschendorf C, Moraes Rodrigues Filho E. High-Flow Nasal Cannula in Critically Ill Subjects With or at Risk for Respiratory Failure: A Systematic Review and Meta-Analysis. Respir Care. 2017;62(1):123-132.

11. Lemiale V, Resche-Rigon M, Mokart D, Pène F, Argaud L, Mayaux J, et al. High-Flow Nasal Cannula Oxygenation in Immunocompromised Patients With Acute Hypoxemic Respiratory Failure: A Groupe de Recherche Respiratoire en Réanimation Onco-Hématologique Study. Crit Care Med. 2017;45(3):e274-e280.

12. Frat JP, Ragot S, Girault C, Perbet S, Prat G, Boulain T, Demoule A, Ricard JD, Coudroy R, Robert R, Mercat A, Brochard L, Thille AW; REVA network. Effect of non-invasive oxygenation strategies in immunocompromised patients with severe acute respiratory failure: a post-hoc analysis of a randomised trial. Lancet Respir Med. 2016;4(8):646-652.

13. Bell N, Hutchinson CL, Green TC, Rogan E, Bein KJ, Dinh MM. Randomised control trial of humidified high flow nasal cannulae versus standard oxygen in the emergency department. Emergency Medicine Australasia. 2015; 27(6):537-541.

14. Frat JP, Thille AW, Mercat A, et al. High-flow oxygen through nasal cannula in acute hypoxemic respiratory failure. N Engl J Med. 2015;372(23):2185-2196.

15. Lemiale V, Djamel M, Julien M, et al. The effects of a 2-h trial of high-flow oxygen by nasal cannula versus Venturi mask in immunocompromised patients with hypoxemic acute respiratory failure: a multicenter randomized trial. Critical Care. 2015;19(1):380.

16. Jones PG, Kamona S, Doran O, Sawtell F, Wilsher M. Randomized Controlled Trial of Humidified High-Flow Nasal Oxygen for Acute Respiratory Distress in the Emergency Department:The HOT-ER Study. Respir Care. 2016;61(3):291-299.

17. Coudroy R, Jamet A, Petua P, Robert R, Frat JP, Thille AW. High-flow nasal cannula oxygen therapy versus noninvasive ventilation in immunocompromised patients with acute respiratory failure: an observational cohort study. Ann Intensive Care. 2016;6(1):45.

18. Milani GP, Plebani AM, Arturi E, et al. Using a high-flow nasal cannula provided superior results to low-flow oxygen delivery in moderate to severe bronchiolitis. Acta Paediatr. 2016;105(8):e368-72.

19. Schmid F, Olbertz DM, Ballmann M. The use of high-flow nasal cannula (HFNC) as respiratory support in neonatal and pediatric intensive care units in Germany - A nationwide survey. RESPIRATORY MEDICINE. 2017 (131): 210-214.

20. Stephan F, Berard L, Rezaiguia DS, et al. High-Flow Nasal Cannula Therapy Versus Intermittent Noninvasive Ventilation in Obese Subjects After Cardiothoracic Surgery. RESPIRATORY CARE. 2017;62(9): 1193-1202.

**Table S2 Quality of the included RCT studies**

| **Study** | **Randomization**  **method** | **Blind**  **method** | **Allocation**  **concealment** | **Withdrawals/**  **Dropouts (NG/NJ)** | **Jadad score** |
| --- | --- | --- | --- | --- | --- |
| **Parke**  **2013** | computer-generated random numbers | Not used | Yes | Yes | 5 |
| **Maggiore**  **2014** | Computer-generated  random numbers | Not used | Yes | Yes | 5 |
| **Corley**  **2015** | Computer-generated  random numbers | Not used | Yes | Yes | 5 |
| **Hernández**  **2016** | Random number  generator | Not used | Yes | Yes | 5 |
| **Futier**  **2016** | Computer-generated  random numbers | Not used | Yes | Yes | 5 |
| **Song**  **2017** | Computer-generated  random numbers | Not used | Unclear | Yes | 3 |
| **Fernandez**  **2017** | Computer-generated  random numbers | Not used | Yes | Yes | 5 |
| **Tiruvoipati**  **2010** | Random number | Not used | Yes | Yes | 5 |
| **Rittayamai**  **2014** | Not mention random method | Not used | Unclear | Yes | 3 |
| **Di mussi**  **2018** | Not used | Not used | Not used | Yes | 1 |

The modified Jadad score was used to evaluate the quality of the included trials. The score awards points for appropriate randomization, presence of concealed allocation, adequacy of double blinding, appropriateness of blinding technique, and documentation of withdrawals and dropouts. The score ranges from 0 to 7, where a score of ≥3 denotes “high quality” based on the original validation studies. Each study was evaluated using the score scale to assess randomization (0-2 points), double blinding (0-2points), Concealment of allocation (0-2points) and withdrawals and dropouts (0-1 point).

**Table S3. Sensitivity analysis of the outcomes**

| **RR (95% CI)**  **Or SMD (95% CI)** | **Postextubation respiratory failure** | **Reintubation** | **Respiratory rate** | **PaO_2_** |
| --- | --- | --- | --- | --- |
| **Sensitivity Analysis** | | | | |
|  | | | | |
| **Change to fixed effect analysis** | 0.64  (0.49,0.83) | 0.54  (0.36,0.80) | -0.72  (-0.96,-0.49) | 0.25  (0.07,0.42) |
| **Change to SJ effect analysis** | 0.60  (0.37-0.96) | 0.58  (0.24-1.38) | -3.0  (-4.7,-1.4) | 5.9  (1.3,10.4) |
| **Change to BT effect analysis** | 0.65  (0.60-0.70) | 0.56  (0.45-0.71) | -3.6  (-4.6,-1.9) | 5.8  (1.9,9.7) |
| **Exclude the high risk of bias study** | 0.62  (0.40, 0.97) | 0.61  (0.29,1.25) | -0.73  (-1.42,-0.03) | 0.20  (-0.07,0.48) |
| **Exclude studies with early determination** | 0.56  (0.33, 0.95) | 0.54  (0.22,1.29) | Na | Na |
| **Exclude any estimated values from included studies** | Na | Na | -0.55  (-1.00,-0.10) | Na |
| **Exclude crossover studies** | Na | Na | -1.12  (-1.45,-0.79) | 0.26  (-0.18,0.70) |

RR:risk ratio; SMD: standardized mean difference; SJ effect analysis: Sidik-Jonkman random-effects model; BT effect analysis: Biggerstaff-Tweedie random-effects model. The SJ and BT effect analysis were calculated with TSA software. RRs and 95% confidence intervals (CIs) were calculated for the binary data, and the standard mean differences (SMDs) and 95% CIs were calculated for the continuous data variables.

**Figure S1. Risk of bias graph: review authors' judgements about each risk of bias item presented as percentages across all included studies.**


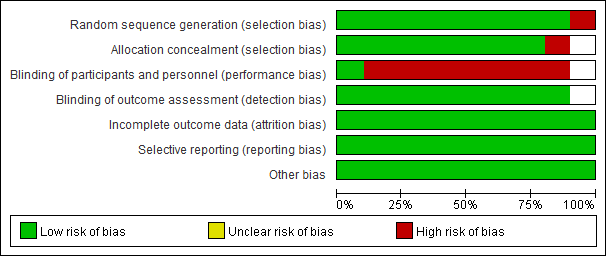


**Figure S2. Risk of bias summary: review authors' judgements about each risk of bias item for each included study.**


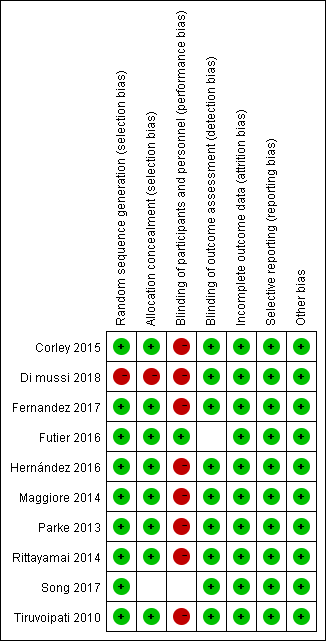


**Figure S3. Comparison of postextubation respiratory failure between the HFNC group and COT group.** Subgroup analysis with regard to HFNC duration (HFNC duration≥24hours or <24 hours).


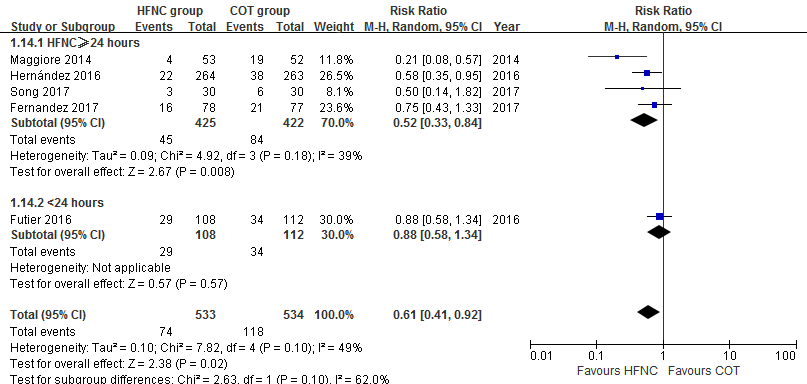


**Figure S4. Comparison of postextubation respiratory failure between the HFNC group and COT group.** Subgroup analysis with regard to HFNC flow rate (HFNC flow≥40L/min or <40L/min).


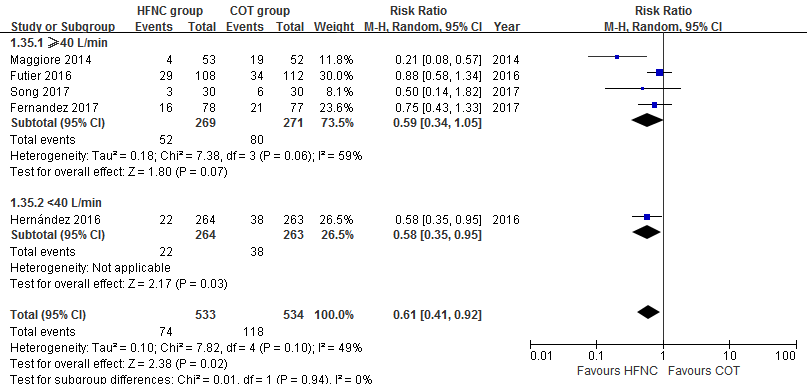


**Figure S5. Comparison of postextubation respiratory failure between the HFNC group and COT group.** Subgroup analysis with regard to severity of patients (Severe subgroup or Non severe subgroup).


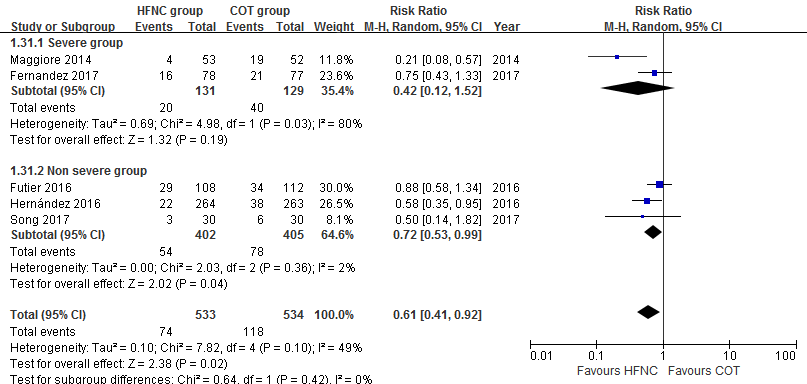


**Figure S6. Comparison of postextubation respiratory failure between the HFNC group and COT group.** Subgroup analysis with regard to hypercapnic or not (Non-hypercapnic subgroup or Mixed patients subgroup). Mixed patients subgroup including hypoxemic or/and hypercapnic patients.


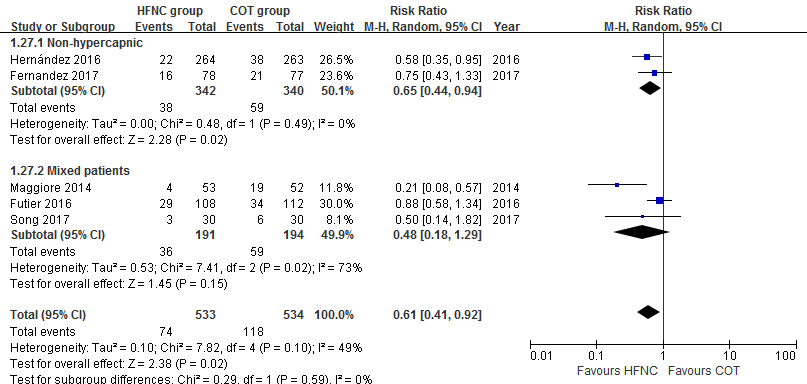


**Figure S7. Trial sequential analysis for comparison of postextubation respiratory failure between two groups.** The blue lines represent the O’Brian-Fleming monitoring boundaries and the futility boundaries. The red line is the cumulative Z-curve. The purple lines are conventional P=0.05 lines. The required sample size for a conclusive result was 1353.


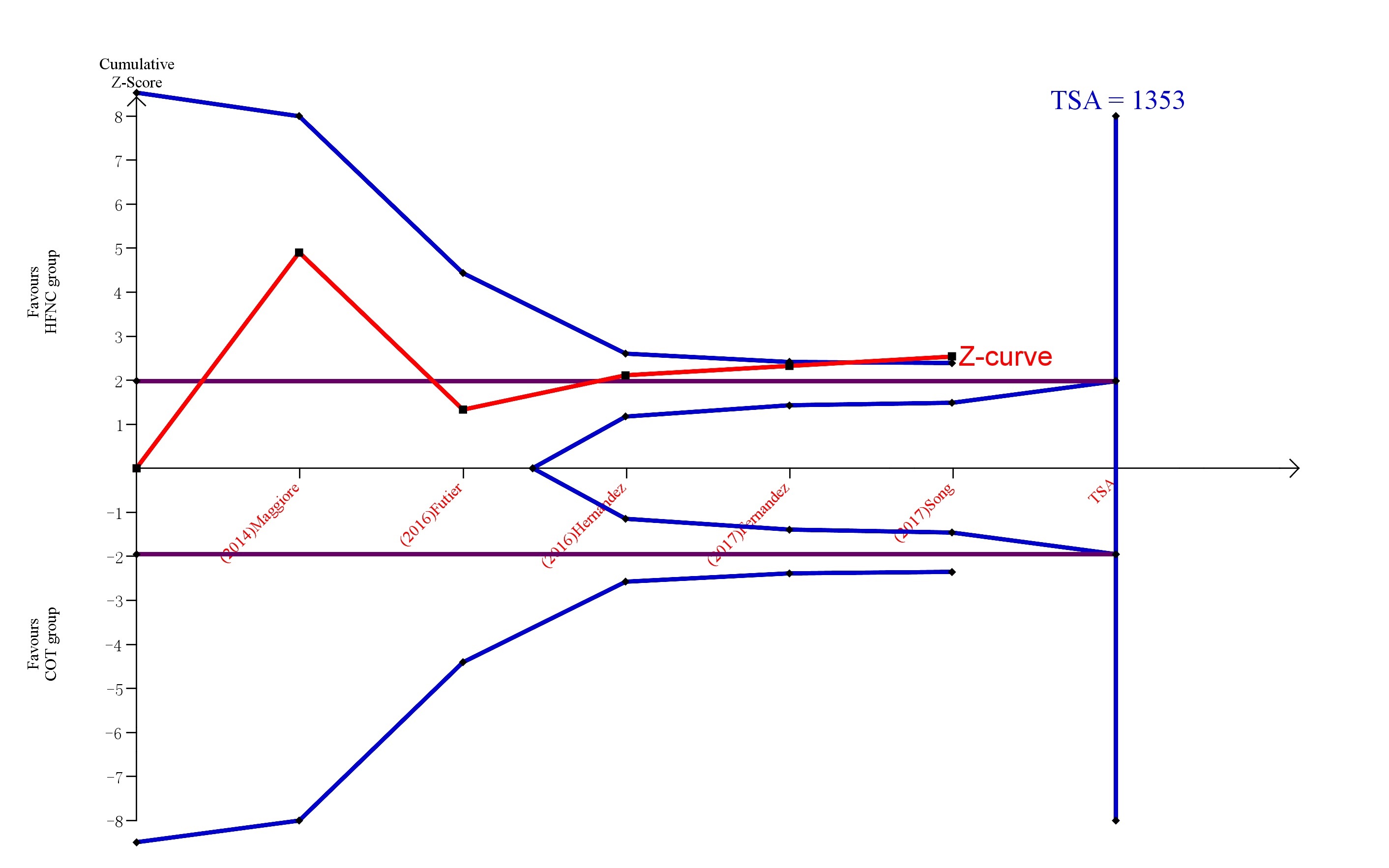


**Figure S8. Comparison of reintubation between the HFNC group and COT group.**


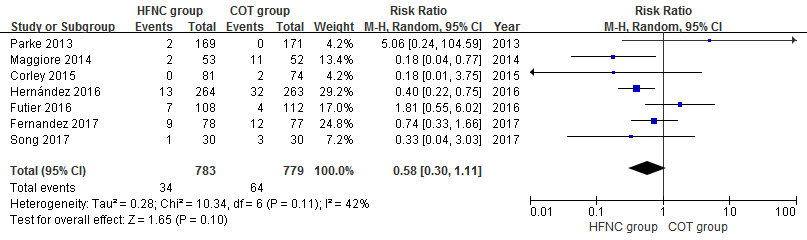


**Figure S9. Comparison of length of ICU stay between the HFNC group and COT group.**


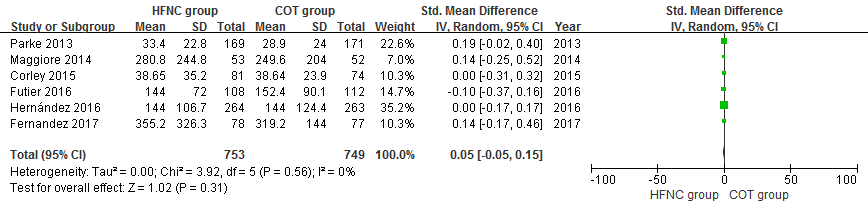


**Figure S10. Comparison of length of hospital stay between the HFNC group and COT group.**


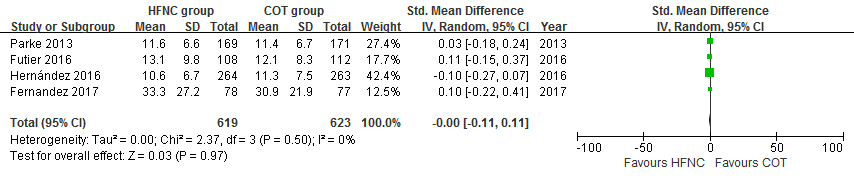


**Figure S11. Comparison of comfort score between the HFNC group and COT group.**


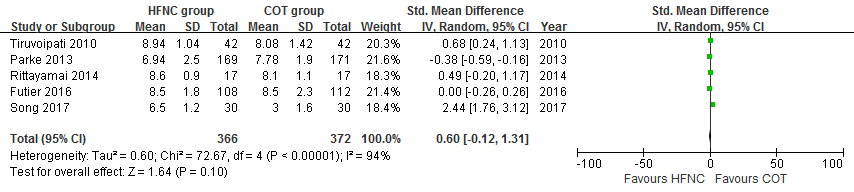


**Figure S12. Comparison of PaCO_2_ between the HFNC group and COT group.**


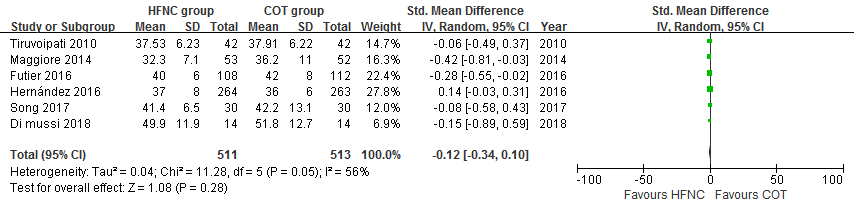


**Figure S13. Comparison of ICU mortality between the HFNC group and COT group.**


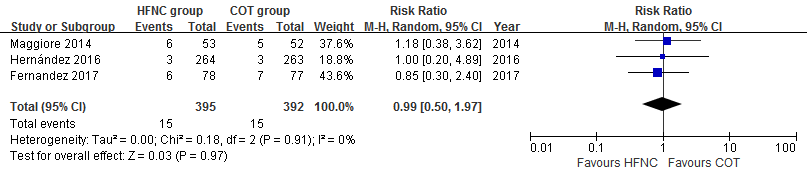


**Figure S14. Comparison of hospital mortality between the HFNC group and COT group.**


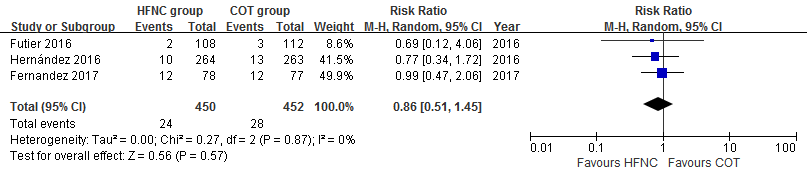


**Figure S15. Funnel plot of comparison for postextubation respiratory failure between HFNC and COT group.**


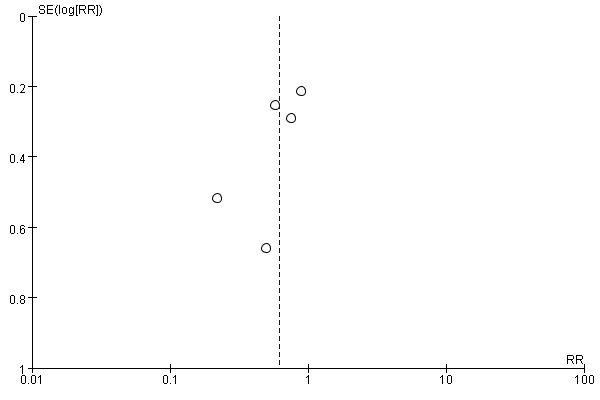


**Figure S16. Funnel plot of comparison for PaO_2_ between HFNC and COT group.**


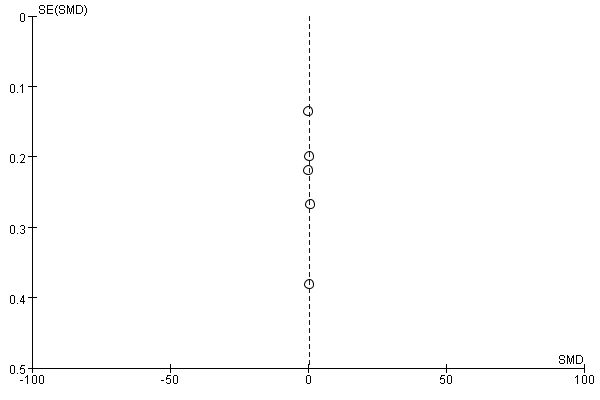


**Figure S17. Funnel plot of comparison for respiratory rates between HFNC and COT group.**


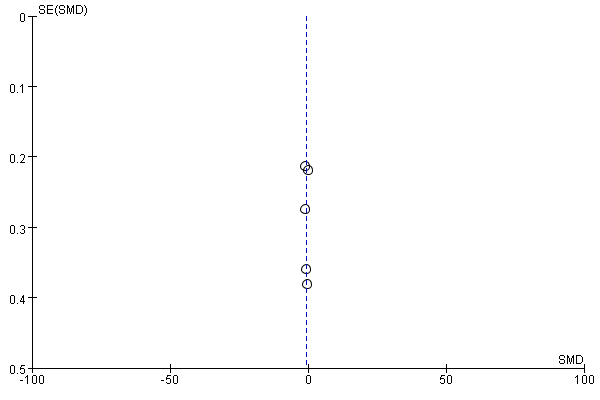


**Figure S18. Funnel plot of comparison for reintubation between HFNC and COT group.**


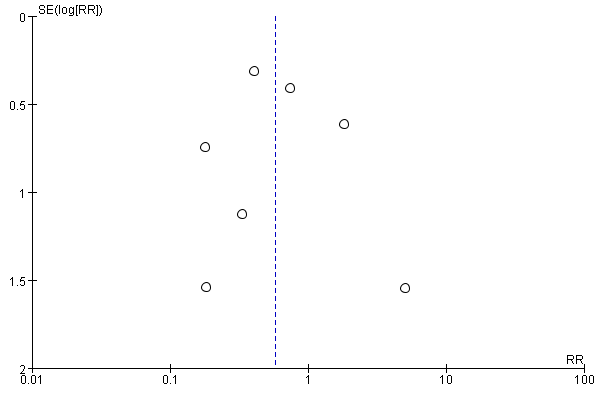


**Figure S19. Funnel plot of comparison for length of ICU stay between HFNC and COT group.**


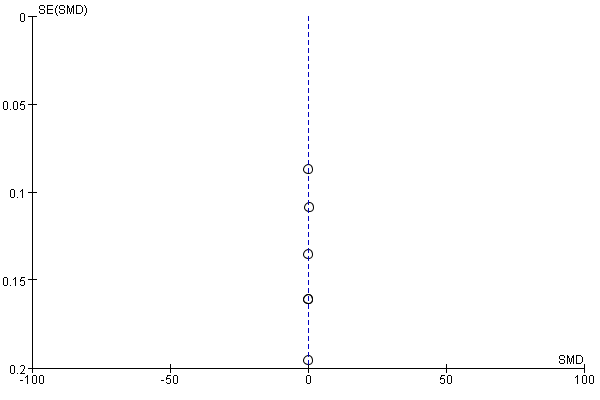


**Figure S20. Funnel plot of comparison for length of hospital stay between HFNC and COT group.**


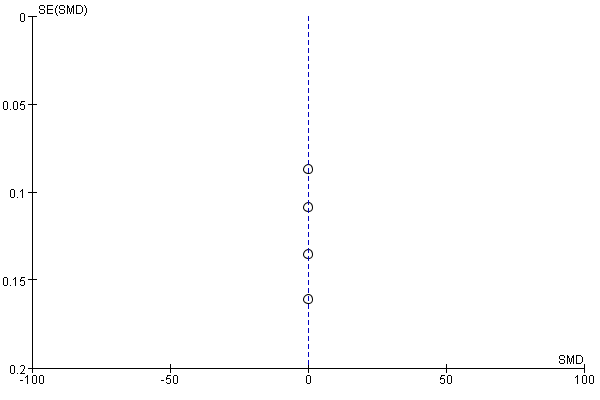


**Figure S21. Funnel plot of comparison for comfort score between HFNC and COT group.**


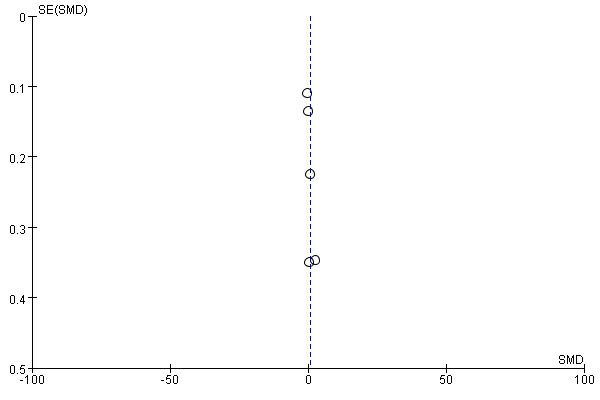


**Figure S22. Funnel plot of comparison for PaCO_2_ between HFNC and COT group.**


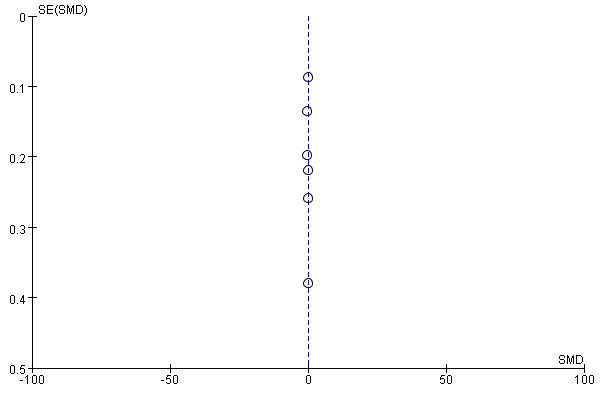


**Figure S23. Funnel plot of comparison for ICU mortality between HFNC and COT group.**


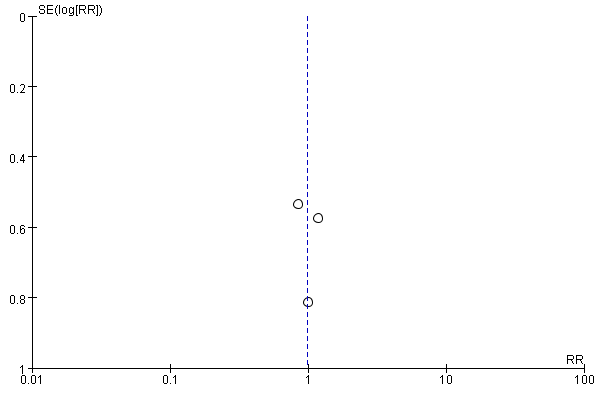


**Figure S24. Funnel plot of comparison for hospital mortality between HFNC and COT group.**


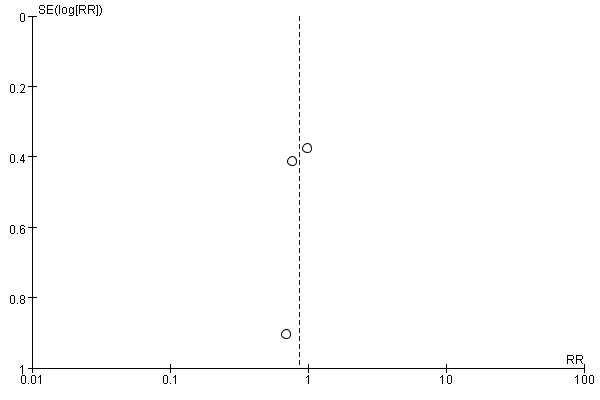

Supplement: Supplementary file 1 — Table S1. Studies Excluded after Full-text Review. Table S2. Quality of the included RCT studies. Table S3. Sensitivity analysis of the outcomes. Figure S1. Risk of bias graph. Figure S2. Risk of bias summary. Figure S3. Subgroup analysis of postextubation respiratory failure between the HFNC group and COT group according to HFNC duration. Figure S4. Subgroup analysis of postextubation respiratory failure between the HFNC group and COT group according to HFNC flow. Figure S5. Subgroup analysis of postextubation respiratory failure between the HFNC group and COT group according to severity of patients. Figure S6. Subgroup analysis of postextubation respiratory failure between the HFNC group and COT group according to hypercapnic or not. Figure S7. Trial sequential analysis. Figure S8. Comparison of reintubation between the two groups. Figure S9. Comparison of length of ICU stay between the two groups. Figure S10. Comparison of length of hospital stay between the two groups. Figure S11. Comparison of comfort score between the two groups. Figure S12. Comparison of PaCO2 between the two groups. Figure S13. Comparison of ICU mortality between the two groups. Figure S14. Comparison of hospital mortality between the two groups. Figure S15. Funnel plot of comparison for postextubation respiratory failure between the two group. Figure S16. Funnel plot of comparison for PaO2. Figure S17. Funnel plot of comparison for respiratory rates. Figure S18. Funnel plot of comparison for reintubation. Figure S19. Funnel plot of comparison for length of ICU stay. Figure S20. Funnel plot of comparison for length of hospital stay. Figure S21. Funnel plot of comparison for comfort score. Figure S22. Funnel plot of comparison for PaCO2. Figure S23. Funnel plot of comparison for ICU mortality. Figure S24. Funnel plot of comparison for hospital mortality. (DOC 613 kb) [file 13054_2019_2465_MOESM1_ESM.doc]
